# Urethane functions can reduce metal salts under hydrothermal conditions: synthesis of noble metal nanoparticles on flexible sponges applied in semi-automated organic reduction†

**DOI:** 10.1039/d2ta09405c

**Published:** 2023-02-24

**Authors:** Olivier Gazil, Johannes Bernardi, Arthur Lassus, Nick Virgilio, Miriam M. Unterlass

**Affiliations:** a Universität Konstanz, Department of Chemistry, Solid State Chemistry Universitätsstrasse 10 D-78464 Konstanz Germany miriam.unterlass@uni-konstanz.de; b CREPEC, Department of Chemical Engineering, Polytechnique Montréal C.P. 6079 Succursale Centre-Ville Montréal Québec H3C 3A7 Canada; c University Service Centre for Transmission Electron Microscopy, Vienna University of Technology Wiedner Hauptstrasse 8-10/137 A-1040 Vienna Austria; d Center for Molecular Medicine of the Austrian Academy of Sciences (CeMM) Lazarettgasse 14, AKH BT25.3 1090 Vienna Austria

## Abstract

We report an additive-free one-pot hydrothermal synthesis of Au, Ag, Pd, and alloy AuPd nanoparticles (NPs) anchored on commercial polyurethane (PU) foams. While unable to reduce the precursor metal salts at room temperature, PU is able to serve as a reducing agent under hydrothermal conditions. The resulting NP@PU sponge materials perform comparably to reported state-of-the-art reduction catalysts, and are additionally very well suited for use in semi-automated synthesis: the NP anchoring is strong enough and the support flexible enough to be used as a ‘catalytic sponge’ that can be manipulated with a robotic arm, *i.e.*, be repeatedly dipped into and drawn out of solutions, wrung out, and re-soaked.

## Abstract 2

Noble metal nanoparticles anchored on flexible polyurethane foams were synthesized by hydrothermal synthesis. Through employing a robotic arm, these ‘catalytic sponges’ were used for organic reductions towards automated lab-scale organic synthesis.

## Graphical abstract

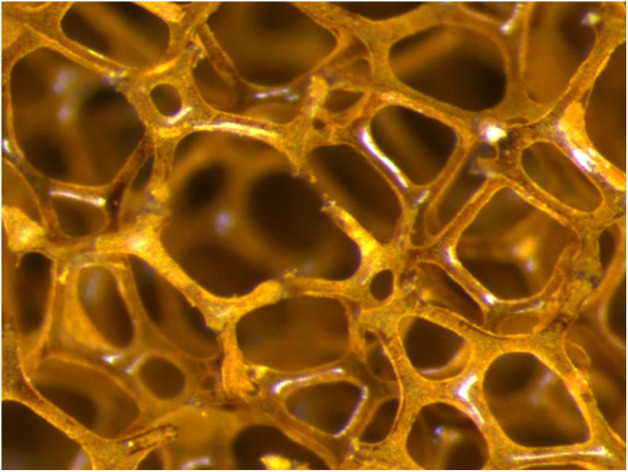

**10th anniversary statement**

The *Journal of Materials Chemistry A* is, in my opinion, the home for both the latest materials developments and in-depth studies of long-standing questions of, *e.g.*, materials synthesis or property mechanisms. I am therefore truly happy to serve as an associate editor for the journal! The breadth of contributions in *J. Mater. Chem. A* reflects one of my favorite characteristics of the community, which is, that we move across traditional disciplinary boundaries – such as inorganic *vs.* organic chemistry – in the quest of finding and making exciting materials for desired applications. I am convinced that materials chemistry will be extremely important in tackling many pressing challenges of our world. As summarized by the UN sustainable development goals (SDGs), we must achieve, *e.g.*, clean water and sanitation (SDG #6), and clean and affordable energy (SDG #7), for every human on this planet. New materials will be key in achieving these goals. We hope that our publications in *J. Mater. Chem. A* have provided a little contribution: they all explore the use of hydrothermal synthesis (HTS) as a green means towards materials. To date, we have contributed examples of inorganic–organic hybrid materials (Leimhofer *et al. J. Mater. Chem. A* 2017; Moura *et al. J. Mater. Chem A* 2022) and of organic polymer networks (Lahnsteiner *et al. J. Mater. Chem A* 2022). With this contribution, we are very excited to share the expansion of the use of HTS to metal nanoparticle–polymer hybrids for application as catalysts.

Metal nanoparticles (NPs) are highly interesting for applications in optics,^1,2^ electronics,^3,4^ imaging and sensing,^5,6^ and catalysis.^6–8^ Au nanoparticles (AuNPs) have been the most exploited in metal NP catalysis,^6–8^ followed by the noble metals Ag,^9^ Pt,^10^ and Pd.^10^ The role of noble metal NPs for catalytic transformations has surpassed initial expectations: C–C cross-coupling reactions (*e.g.*, Sonogashira, Heck, or Kumada couplings), for example, which classically rely on homogeneous, molecular Pd complexes, are now known to profit, in part, from catalytically active PdNPs that form *in situ*.^11,12^ Mechanistically, noble metal NP catalysis functions by surface processes: reactant molecules are adsorbed on the NPs' surfaces by coordination to unsaturated surface atoms, through which to-be-broken bonds within the reactants are weakened.^13^ The smaller a NP, the higher its amount of surface atoms, and hence the better its catalytic activity. For instance, bulk Au is catalytically inert, but becomes active when downsized to the nanoscale,^14,15^ specifically <20 nm.^7^ Noble metal NPs are mostly made by bottom-up techniques, typically by reducing metal salt precursors in solution.^16^ Standard reducing agents comprise H_2_, formaldehyde, citrates, CO, hydrazine, NaBH_4_, and alcohols.^16,17^ Many of these chemicals pose significant risks to human health and the environment.^18,19^ To prevent both NP growth beyond desired sizes and aggregation, organic molecules that cap the NP surfaces are often added to their syntheses. However, capping typically leads to decreased catalytic activity (inaccessibility of NP surfaces for reactant molecules).^20^ For actual application of metal NP catalysis, the NPs have to be anchored on a support. While the catalytic transformation would still work with NPs in dispersion, their recovery is problematic: due to the small particle sizes, advanced techniques, such as ultracentrifugation, must be used. Supports can be subdivided into two broad classes: (i) mechanically rigid supports such as metal oxides (*e.g.*, TiO_2_, CeO_2_, Al_2_O_3_, or SiO_2_),^21–24^ and (ii) mechanically soft supports. The latter, sometimes referred to as “soft structured catalytic supports” (S2CS) or simply “sponges”, have only recently begun to be exploited.^25,26^ In industrial continuous-flow catalysis (performed in packed bed reactors), significant pressures and flow rates exist, requiring high mechanical strength, which only rigid supports can provide. For instance, NPs anchored on soft hydrogel supports were tested in flow-through catalytic reactors, and reported to not sustain beyond *ca.* 100 kPa of pressure drop^27^ – significantly below the industrially relevant range of typically 1000–3000 kPa.^28^

In this work, we have set out to (i) synthesize noble metal NPs on soft deformable supports, while (ii) maximally adhering to the principles of green chemistry, and (iii) testing the resulting materials as reusable catalysts of potential interest for semi-automated organic synthesis. Our strategy towards these goals was as follows: first, we wanted to avoid harmful solvents and additives (reductants and capping agents). Second, instead of synthesizing flexible foams as sponges, we aimed at using commercial ones. Third, we wanted to assess their usefulness as catalyst materials for automated synthesis by performing several cycles, and by employing a low-cost robotic arm. Parts of our approach have been addressed to varying depth in the literature individually, as briefly summarized in the following.

We decided to generate the NPs by hydrothermal synthesis (HTS). HTS employs liquid superheated water (aka high-temperature water, HTW) as the reaction medium.^29^ As H_2_O is one of the safest and least harmful solvents, the technique is considered green. Synthesis in HTW is subdivided into three regimes: the hydrothermal (broadly 100 °C < *T* ≤ 250 °C, more typically 150 °C < *T* ≤ 250 °C), the near-critical (250 ≤ *T* ≤ 374 °C), and the supercritical regime (*T* > 374 °C).^30^ HTW bears the potential of a near-universal reaction medium: various metal oxide compounds (*e.g.*, quartz, sapphire, zeolites),^31,32^ carbon materials,^33^ and well-defined organic molecules (*e.g.*, rylene imides, quinoxalines, perinone)^34–36^ have been generated by HTS. Beyond covalent bond formation, HTS has also been used to generate metallic bonds, especially towards NPs. Examples include Ir and Ir–Pd NPs (180 °C, reductant: NaBH_4_),^37^ AuNPs (110 °C, reductant: trisodium citrate, capping agent: CTAB),^38^ or AgNPs (100–180 °C, reductant: sodium alginate).^39^ Furthermore, AgNPs, CuNPs and ZnNPs have been synthesized in supercritical HTW in continuous flow (*T* ≥ 400 °C, for AgNPs no additives or with polyvinylpyrrolidone as the additive was used).^40,41^ HTS of (noble) metal NPs is in fact geomimetic: geological Au deposits are naturally found as hydrothermal ore deposits, with Au^0^ precipitating from aqueous chloro and hydrogensulfido aurate solutions (*e.g.*, [AuCl_2_]^−^_(aq)_, [Au(HS)_2_]^−^_(aq)_).^42^ All the latter examples of HTS of noble metal NPs, except for synthesis in supercritical water, still employ added reducing agents. In contrast, we aim in this work at directly using the support itself as a reducing agent. Very interestingly, there are two subfields in the literature that seem disconnected. On one side, various studies report the preparation of metal NPs anchored on polysaccharide supports for catalysis applications, but these materials are in the majority of cases prepared with the addition of a reducing agent (*e.g.*, summarized in a review paper on supported PdNPs for catalysis by Wolfson & Levi-Ontmann).^43^ On the other side, the thriving field of ‘biosorption’ aims at recovering (precious) metal ions from waste waters using biopolymer sorbents (mainly polysaccharides and lignocellulosic biomass). The biopolymers serve as both sorbent and reductant, and indeed metal NPs anchored on the biosorbent are obtained (*cf.* a recent comprehensive review by Hunt and coworkers).^44^ Yet, these materials are typically subsequently treated with concentrated acids (*e.g.*, HNO_3_) to recover the metals, but are not used in added value applications.^44^ The few examples of supported NP synthesis (without an additional reducing agent) used for catalysis comprise a report by Wu *et al.* on the synthesis of PdNPs on nanocellulose as both reductant and support (synthesized in pH = 1 aqueous HCl at *T* = 100 °C for 12 h),^45^ and a report by Chen *et al.* on the synthesis of bimetallic AuPd NPs on graphene nanosheets as both reductant and support (synthesized in water at room temperature (rt) for only a few minutes).^46^ However, the employed nanoscopic supports are difficult to re-use (as described earlier: methods like ultracentrifugation would be necessary), and, as Hunt and coworkers have pointed out, even non-nanoscopic biopolymer supports bear the downside of “[…] poor mechanical strength and small particle size, resulting in difficulties using it (sic) in batch and continuous systems.”^44^ Consequently, the reported supported NP catalysts tested for use as, *e.g.*, redox catalysts have not been investigated with respect to actual re-use.^45,46^‡‡Note that some supported NP materials are described as being studied in “reuse over several cycles”, however, the materials are not actually re-used, but further substrate is added to the reaction mixture, once all substrate is consumed.^48^ Such approaches do therefore not probe re-useability. To address this shortcoming, we decided to employ commercial polyurethane foams (PUFs), *i.e.*, sponges. These are low-cost, readily available in any supermarket, and, most importantly, they have been industrially optimized for good mechanical performance and chemical stability on repeated use: dish washing sponges are able to undergo thousands of soaking and squishing cycles while in contact with hot water and a wide range of compounds (fats, acids, surfactants, *etc.*). Several studies by Edouard, Jierry, Ritleng and coworkers used modified PUFs (surface coverage with polydopamine or polydopamine and AgNPs) as reusable catalysts (5–6 cycles) for the oxidation of dyes (methylene blue, azo dyes),^25,26^ and Macanás and coworkers reported modified AgNP decorated PUFs as re-useable redox catalysts.^47^‡ In both cases, reducing agents (polydopamine^26^ and NaBH_4_,^47^ respectively) were added to synthesize the NPs anchored on PUF. We are not aware of any report that directly employs PUFs as both support and reducing agent. Yet, we hypothesized that the NH(C=O) part of the urethane (aka carbamate) function would be able to act as a reducing agent towards noble metal NPs. Our hypothesis was based on dimethylformamide (DMF) being a well explored reducing agent of Ag and Au salts.^48^ Furthermore, we speculated that the urethane function would also help to anchor the NPs, *i.e.*, through coordination to lone pairs of N and O atoms.

## Results and discussion

**Fig. 1:**
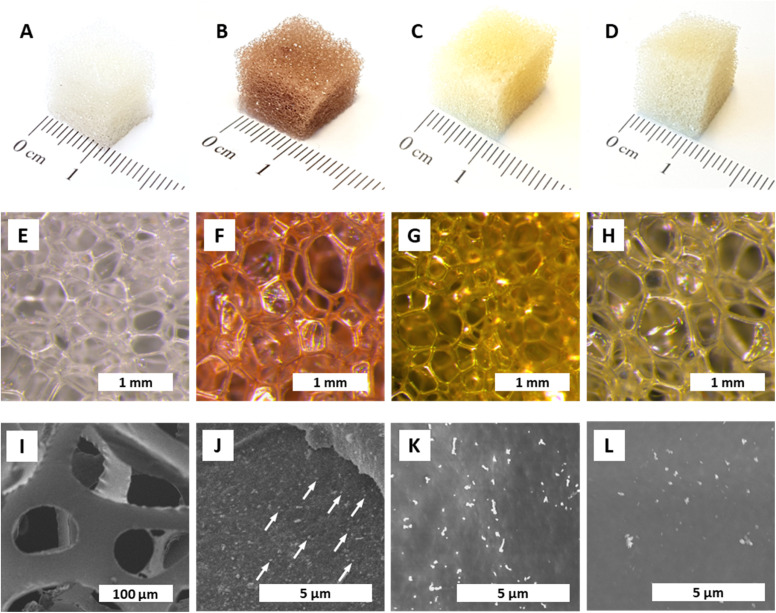
Aspect and morphology of NPs@PUF. All specimens were synthesized at *T*_R_ = 120 °C, *t*_R_ = 3 h, and [*M*^*x*+^] = 0.25 mM. Macroscopic aspect (A–D) and optical micrographs (E–H) of pristine PUF and NPs@PUFs: pristine PUF (A and E), AuNP@PUF (B and F); AgNP@PUF (C and G), PdNP@PUF (D,H). SEM micrographs of pristine PUF(I), and NPs@PUFs: AuNP@PUF (J) with some AuNPs indicated with white arrows; AgNP@PUF (K); and PdNP@PUF (L).

In a first set of qualitative experiments, monitoring of the formation of AuNPs decorating polyurethane foams (AuNPs@PUF) was performed at reaction temperatures *T*_R_ of 25 °C (rt), 100 °C, and 120 °C. Synthesis at 120 °C was performed inside a glass vial placed in a microwave (MW) reactor (see the ESI† for details). An autogenous pressure of *ca.* 3 bar arises at 120 °C, and real-time observations are possible *via* a camera filming the glass vial. In a typical experiment, a cube (1 cm edge length) of polyurethane foam (PUF, purchased from a local branch of the hardware store OBI, see the ESI†) was plunged in 5 mL of an aqueous solution of HAuCl_4_ (0.25 mM) and was stirred at the specified *T*_R_. The synthesis was stopped when a change in color was no longer observed. The starting PUF cube (Fig. 1A) is white and the aqueous HAuCl_4_ solution is translucent light yellow. A change in color is indicative of NP formation, as noble metal NPs display strong absorption bands in the visible spectrum. For AuNPs, a strong plasmon band at ∼500 nm^49^ is responsible for the sponge turning red upon AuNP formation (Fig. 1B). With proceeding AuNP formation, the PUF cube turned increasingly red, and a synthesis was judged as having reached maximal conversion when the red color would not further intensify. To check if the reaction was complete, as indicated by the cessation of color change, the strong reducing agent NaBH_4_ was added to the solution. Any change of color or precipitation indicated leftover [AuCl_4_]^−^, hence demonstrating an incomplete reduction by the PUF. These first qualitative experiments evinced that the syntheses at *T*_R_ = 25 °C, 100 °C, and 120 °C required, respectively, one day, 30 minutes, and *ca.* 3 minutes for completion (consumption of all AuCl_4^−^_).

In the same way, the reductions of AgNO_3_ solutions to AgNPs and of Na_2_PdCl_4_ solutions to PdNPs were probed. Colorwise, the formation of AgNPs and PdNPs, respectively, is suggested by the change of the colorless PUF cube to yellow (Ag: plasmon band at ∼400 nm)^49^ and gray (Pd NPs are black). In contrast to AuNPs, AgNPs did not form at rt, only marginally at *T*_R_ = 100 °C, and partially at 120 °C. For PdNPs, an incomplete reaction to varying degrees was observed at all tested *T*_R_. The AgNPs@PUF sponges are yellow (Fig. 1C), as expected from their plasmon absorption bands, while PdNPs@PUF sponges exhibit light brown color (Fig. 1D), reminiscent of the color of the precursor Na_2_PdCl_4_, instead of the expected black/gray. This points at the incomplete reduction of Pd^2+^, which is in line with our observation that additional Pd^0^ formed in solution in all cases after the addition of NaBH_4_, after stopping the synthesis reaction. After this initial screening, the HTS conditions were set to *T*_R_ = 120 °C, *t*_R_ = 3 h, and [*M*^*x*+^] = 0.25 mM. All subsequently discussed analytical and performance data were collected from specimens synthesized under these set conditions.

The colors of the NP@PUF materials are well reflected in their optical micrographs displayed in Fig. 1E–H. Aside from optical microscopy, we performed scanning electron microscopy (SEM) to further study the NP@PUF materials' morphologies. Fig. 1I–L show SEM images of the pristine PUF (Fig. 1I), AuNPs@PUF (Fig. 1J), AgNPs@PUF (Fig. 1K) and PdNPs@PUF (Fig. 1L). From the SEM image of the pristine foam, the open cell structure and the smooth surfaces of the cell walls are clear (see Fig. S1† for higher magnification). SEM of the AuNPs@PUF evinces the presence of nanosized particles of homogeneous sizes and distribution on the PUF cell walls (some AuNPs are indicated by white arrows in Fig. 1J). Similar observations are made for AgNPs@PUF and PdNPs@PUF, but the particles are brighter in SEM compared to AuNPs.

**Fig. 2:**
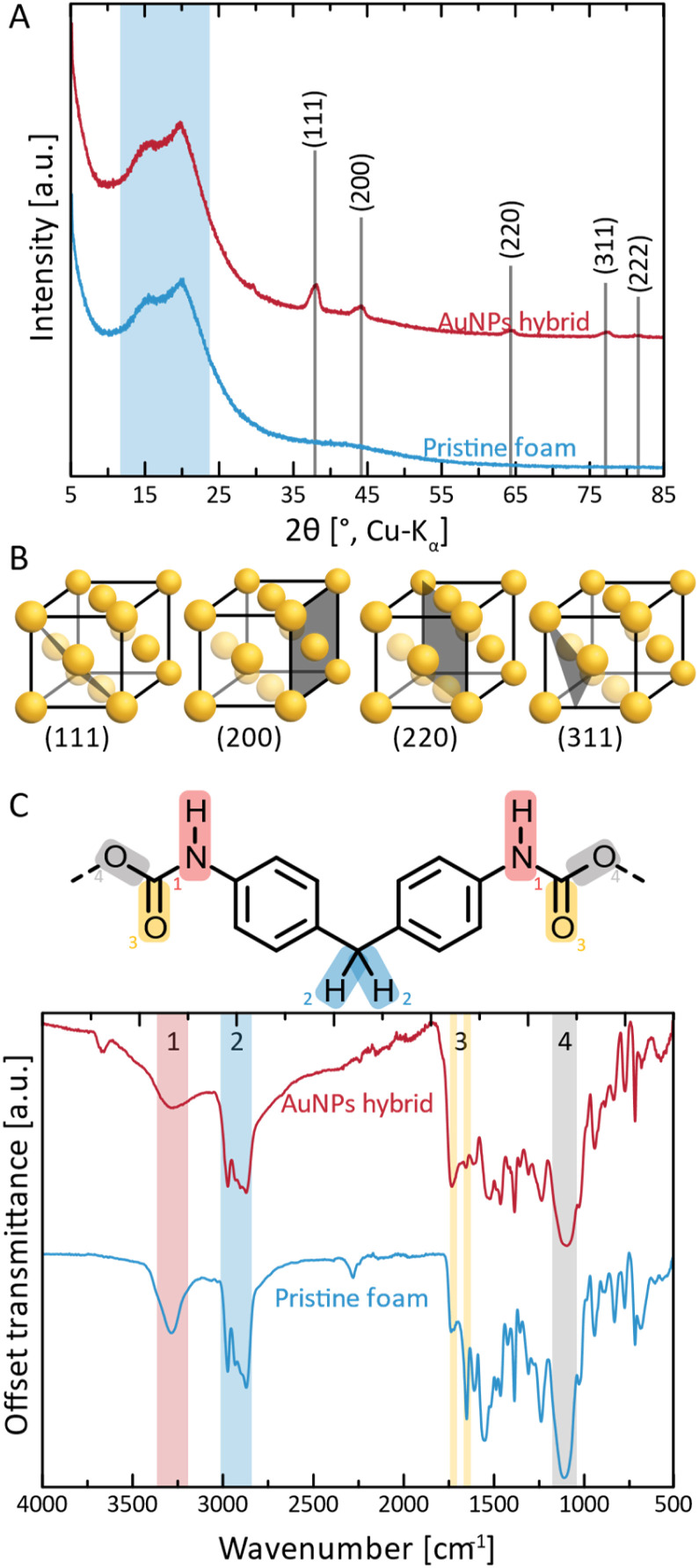
Structural and chemical characterization of AuNPs@PUF and pristine PUF. (A) Diffractograms of AuNPs@PUF (red curve) and pristine PUF (blue curve); (B) reflections associated with the crystal structure of elemental Au (fcc) are labelled (*hkl*), and the corresponding lattice plane in the structure is illustrated. (C) ATR-FT-IR spectra of AuNPs@PUF (red curve) and pristine PUF (blue curve); also shown is an excerpt of the structure of methylene diphenyl diisocyanate-based PU, with functional groups highlighted as (1) in red: urethane N–H; (2) in blue: methylene –CH_2_–; (3) in yellow: urethane CO; and (4) in gray: urethane –O–.

Next, we performed structural characterization of all three NPs@PUF materials, as well as pristine PUF for reference, *via* powder X-ray diffraction (PXRD), see Fig. 2A. Pristine PUF (blue curve, Fig. 2A) displays an amorphous halo in the region of *ca.* 10–25° (2*θ*, Cu-K_α_) with two maxima centered around *ca.* 15.8° and *ca.* 20.4° (2*θ*, Cu-K_α_), respectively (highlighted by the blue background in Fig. 2A). The diffractogram is in full agreement with reported PXRD data of PU.^50^ The diffractogram of AuNPs@PUF (red curve, Fig. 2A) features the PUF's amorphous halo, and, additionally, several reflections characteristic of elemental Au. Au^0^ crystallizes in the face-centered cubic (fcc) structure (displayed in Fig. 2B),^51^ and the corresponding reflections are present, namely at 38.1 (111), 44.3 (200), 64.6 (220), 77.7 (311) and 81.1° (2*θ*, Cu-K_α_) (222). The reflections are relatively broad, which is typical of NPs. From PXRD data, we determined the average Au nanocrystallite size *via* both the Scherrer formula (ESI, eqn (S1)†), using the full width at half maximum (FWHM), and Rietveld's method (ESI). Scherrer's method yields an average crystallite size of 11.9 nm, while Rietveld's method yields an average size of 14.5 nm. Ag^0^ also crystallizes in fcc, and hence the same (*hkl*) are expected, albeit at slightly smaller 2*θ* values (lattice parameters: *a*(Au) = 4.07 Å, *a*(Ag) = 4.09 Å). Indeed, AgNPs@PUF diffractogram (Fig. S2A†) is very similar to that of AuNPs@PUF. In contrast, the diffractogram of PdNPs@PUF does not show Bragg reflections at all (see ESI,† Fig. S2A). Pd^0^ also crystallises in fcc (*a*(Pd) = 3.88 Å), hence, a diffractogram similar to AuNPs@PUF and AgNPs@PUF would be expected. The absence of Bragg reflections for PdNPs@PUF points at either the formation of amorphous PdNPs or of very small NPs (≲5 nm).

Next, the chemical structure of the NPs@PUF materials, as well as the chemical interactions between metal NPs and the PUF support, were analyzed by attenuated total reflectance Fourier transform infrared (ATR-FTIR) spectroscopy, *cf.*Fig. 2C. PUs are synthesized by the polycondensation of diisocyanate with di- or polyol monomers, generating the urethane (aka carbamate) linking function. In Fig. 2C, an excerpt of the PU structure relevant to this study is shown, namely the part of the repeating unit arising from the diisocyanate monomer. In the present case, it becomes clear from the ATR-FTIR spectra that the diisocyanate component used to generate the used PUF must have been methylene diphenyl diisocyanate (MDI) given that the ATR-FTIR spectra perfectly fit the ones found in the literature.^52^ Modes characteristic of four functional groups in the PUF can be clearly observed. First, region #1 (red, Fig. 2C), corresponds to urethane N–H stretching and is found at ∼3275 cm^−1^. The intensity of this mode is decreased in AuNPs@PUF compared to pristine PUF, and additionally, the mode is wider in AuNPs@PUF compared to pristine PUF. Both changes are indicative of a possible interaction of the N–H function with AuNPs, or at least a decrease in H-bonding within the PUF component caused by the AuNPs' presence. It is noteworthy that the used ATR variant of infrared spectroscopy has a typical penetration depth of *ca.* 1 μm, making it rather a surface analysis technique. As the AuNPs are located at the surface of the PUF, the interactions between AuNPs and PUF should be especially well visible *via* ATR-FTIR analysis. The next region, highlighted in blue (#2 in Fig. 2C), corresponds to characteristic C–H stretching modes (∼2975 cm^−1^ and ∼2870 cm^−1^), arising from the PUF backbone, *i.e.*, from both MDI and diol parts of the repeating unit (diol segments not illustrated in Fig. 2C). The most interesting peaks are those of region #3 (yellow, Fig. 2C), corresponding to C=O modes (1640 cm^−1^ and 1720 cm^−1^), as they are related to the NPs' reduction and stabilisation. The relative intensity of these modes gets inverted, *i.e.*, the 1st C=O peak (1720 cm^−1^) has a smaller intensity compared to the 2nd peak (1640 cm^−1^) for the pristine PUF, while the opposite is observed for the AuNPs@PUF. This is likely due to a decrease in H-bonding to C=O in the PUF caused by the AuNPs. More precisely, H-bonding is known to decrease the intensity of C=O modes.^53^ The pristine PUF, rich in H-bonding, has a weak 1720 cm^−1^ peak, but a stronger one at 1640 cm^−1^. Interactions between AuNPs and carbonyl groups weaken the C=O groups' abilities to accept H-bonds, as part of their lone pairs is now used to coordinate to Au surface atoms, leading to a more intense IR absorption at 1720 cm^−1^, and a less intense absorption for the 1640 cm^−1^ mode. These observations are in line with C=O groups' strong ability to coordinate to metals.^51,54,55^

For both AgNPs@PUF and PdNPs@PUF, more intense modes at ∼1720 cm^−1^ (Fig. S2B†), compared to the pristine PUF, suggest that AgNPs and PdNPs are also bound to the PUF support *via* C=O functions. However, the effect is less pronounced for AgNPs@PUF and PdNPs@PUF than for AuNPs@PUF. This might be due in part to the enhancement effect that AuNPs have on IR absorption (linked to the field enhancement properties of surface plasmon resonance), as for instance exploited in surface-enhanced infrared absorption spectroscopy (SEIRAS).^56^ Moreover, it could also be that the C=O modes' intensity changes are less pronounced for the AgNPs@PUF and PdNPs@PUF samples, as there are simply less NPs present in both systems (see the discussion about thermogravimetric analysis (TGA) results). Lastly, we assign region #4 (gray, Fig. 2C) to either/both the urethane C–O stretching (1100 cm^−1^) or/and to the siloxane functional group, Si–O–Si. The presence of Si–O–Si is conceivable, as (i) siloxane-based additives are sometimes used in industrial PU formulations (as surfactants for improving pore formation and uniformity of PUFs),^57^ and (ii) HTW is known to partly dissolve SiO_2_ from glass vessels in the form of silicic acid species that reprecipitate as nanosilica once HTW is cooled back to ambient H_2_O.^58^ Note that later discussed EDX analysis (see the ESI†) also evinced the presence of Si.

**Fig. 3:**
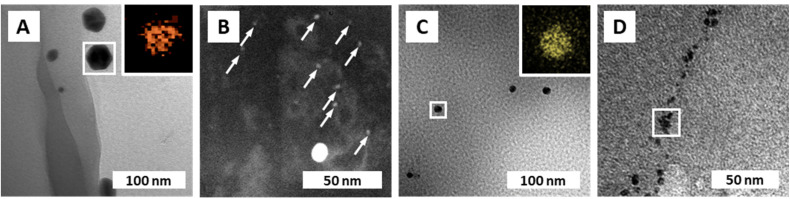
NP composition and morphologies. (A) TEM micrograph of AuNPs@PUF with EDX mapping (inset) of the framed region, confirming the presence of Au. (B) Dark-field STEM micrograph of AuNPs@PUF displaying the presence of a second population of small AuNPs (indicated with white arrows), referred to as gold “seeds”. (C) TEM micrograph of AgNPs@PUF with EDX mapping (inset) confirming the presence of Ag. (D) TEM micrograph of PdNPs@PUF with EDX spectrum of the framed region displayed in ESI† Fig. S3.

**Fig. 4:**
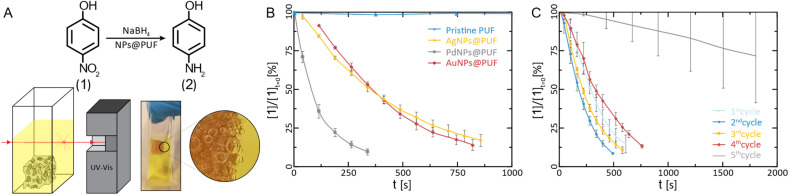
Catalytical characterization of AuNPs@PUF. (A) Reaction pathway for the reduction of 4-NP to 4-AP alongside the schematic representation and photograph of the UV-vis catalysis setup. Note in the magnified view, the presence of H_2_ bubbles in the PUF. Reduction kinetics of 4-NP: (B) plot of the relative concentration of 4-NP *versus* time for the determination of the reaction rate constant (*N* = 3) and; (C) reaction kinetics *versus* the number of completed reduction cycles with AuNP hybrids (*N* = 3), showcasing their reusability potential.

Transmission electron microscopy (TEM) was used to further characterize the NPs' size (*via* image analysis, see the ESI†), shape, and chemical composition (*via* energy dispersive X-ray spectroscopy; EDX). From SEM, it became clear that the PUF support has an open-cell foam structure with cell wall thicknesses of ≈60 μm (Fig. 1I). We suppose that in NPs@PUF, the NPs are located on the PUF cell wall surfaces. For AuNPs@PUF, the TEM micrographs reveal two AuNP populations (Fig. 3A&B): fully grown particles of ∼30 nm in diameter (Fig. 3A), and “seeds” of ∼3 nm in diameter (Fig. 3B). The larger AuNP population (Fig. 3A) shows faceted particles of isometric crystal habit. We hypothesize that the bimodal size distribution is linked to the growth difference between a nucleus growing unrestricted on the polymer fiber surface and another one that would be spatially restricted within the PUF, *i.e.*, in between the PU polymer chains.^59,60^ For AgNPs, only one NP population is observed in TEM (Fig. 3C), with an average size of *ca.* 16 nm. Finally, PdNPs' TEM analysis (Fig. 3D) evinces very small NPs (∼5 nm). This could explain the absence of Bragg reflections in the diffractogram of PdNPs@PUF (ESI,† Fig. S2A; *cf.* previous discussion of PXRD of all materials).^61^ Two other possible explanations are, (i) that the PdNPs are Pd^0^, but amorphous (amorphous–crystalline Pd^0^ heterostructures have been reported^62^), or (ii) that the NPs are not composed of pure Pd^0^. The EDX spectrum associated with the PdNPs@PUF TEM analysis reveals that chlorine and palladium are both present (ESI,† Fig. S3). This lets us hypothesize that the precursor used here, Na_2_PdCl_4_, when dissolved in H_2_O, must result in different complexes such as [PdCl(H_2_O)_3_]^+^ or [PdCl_2_(H_2_O)_2_], precipitating as mixed NPs of Pd^0^ and a PdCl_2_-type phase onto PUF. PdCl_2_'s bonding has a partly covalent character, and its two polymorphs are built up of PdCl_2_ chains and Pd_6_Cl_12_ clusters, respectively.^63,64^ Hence, amorphous forms are conceivable. Also, the crystallinity of Pd^0^ is not likely in such small NPs if significant amounts of foreign species (such as the speculated PdCl_2_-type species or other) are present. Overall, at this point, we cannot tell if the PdNPs appear amorphous in PXRD because of their size, because they are actually amorphous, or, because they contain some amount of PdCl_2_-type phases. In any case, the presence of Cl atoms in EDX strongly suggests that Pd^2+^ has not been fully reduced to Pd^0^ by the applied HTS. Consequently, the PdNPs@PUF cannot be employed for catalysis without an additional reduction step. This step was realized simultaneously with our model reduction reaction, *i.e.*, the transformation of 4-nitrophenol (1, Fig. 4A) into 4-aminophenol (2, Fig. 4A) using sodium borohydride (NaBH_4_) as the reducing agent.

The reduction of 1 to 2 using NaBH_4_ is a standard model reduction to evaluate the performance of reduction catalysts. Despite being a fairly strong reductant, NaBH_4_ is, at rt, not able to reduce *p*-nitrophenol to *p*-aminophenol without the presence of a catalyst. Furthermore, 1's reduction to 2 can be conveniently monitored by UV-vis spectroscopy, which we also applied here: the relative concentration of 1 ([*1*]/[*1*]_*t*=0_) can be assessed *via* the relative intensity of the absorbance (*A*/*A*_*t*=0_) at 400 nm.^9,65^ We performed these experiments by immersing the NPs@PUF materials directly in an aqueous solution of 1 and NaBH_4_ inside a UV-vis spectroscopy cuvette (Fig. 4A). The apparent reaction rate constant, *k*_app_, was calculated by fitting an exponential curve to the relative concentration of 1*versus* time (Fig. 4B). The first few points were excluded from the fitting because of the induction time observed in the reaction. Indeed, dissolved oxygen causes a side reaction (*i.e.*, the conversion of 2 back to 1),^66^ which was reduced to a minimum by purging with nitrogen before the reaction. As expected, the pristine foam is not able to achieve the reduction of 1 (*k*_app_ ≈ 0, Fig. 4B). AuNPs@PUF and AgNPs@PUF display similar apparent reaction rates of 3.08 × 10^−3^ s^−1^ and 2.73 × 10^−3^ s^−1^, respectively (see Table 1). However, PdNPs@PUF performs differently with *k*_app_ = 9.65 × 10^−3^ s^−1^ (Fig. 4B and Table 1). Hence, the reaction seems to be significantly more efficient in the presence of PdNPs@PUF than in the presence of AuNPs@PUF and AgNPs@PUF. This is likely due to the smaller size of PdNPs: it is well-known that smaller NPs are more catalytically active compared to larger ones, due to their higher surface atom/bulk atom ratio.^67^ Furthermore, as discussed earlier, ATR-IR spectroscopy data have pointed at bonding between the NPs and the PU backbone. Consequently, one might suppose that synergistic or antagonistic interactions between the support and NPs are of relevance to their catalytic performance. Reduction-rate altering effects rooted in NP–support interactions have been found between, *e.g.*, nitrogen-containing organic polymers and metal NPs.^68^ However, we can at this point neither confirm nor deny the presence of such effects in the NP@PUF materials.

**Table 1:** Key parameters quantifying the catalytic properties of NPs@PUF hybrids

	Average NP diameter *d* (nm)	% *m*/*m* NPs^a^ (%)	Reaction rate *k*_app_ (s^−1^)	Normalized reaction rate *k*′_app_ (s^−1^ m^−2^ L)
AuNP hybrid	3 ± 0.3	0.68 ± 0.01	3.08 × 10^−3^	4.43 × 10^−4^
AgNP hybrid	16 ± 3	0.3 ± 0.1	2.73 × 10^−3^	3.49 × 10^−3^
PdNP hybrid	5 ± 2	0.3 ± 0.1	9.65 × 10^−3^	4.05 × 10^−3^

a Weight percentage corresponding to the mass of NPs per total mass of NPs@PUF.

Both the open-cell architecture of PUFs, reflected in the photographies as well as the optical micrographs of the specimen (Fig. 1), and the uniform loading with metallic NPs, reflected in SEM images, are promising features for catalysis applications. Yet, PUFs are known to depict relatively poor wettability,^69^ which can impede the catalytic reduction of substrates in aqueous solution. Indeed, we observed an accumulation of gaseous H_2_ (generated from NaBH_4_) within the foam during the reaction (*cf.* photograph in Fig. 4A). Hydrogen remains trapped and slows down the reduction of 1, if not properly removed *via* manual compression of the NPs@PUF materials. Compressing the materials has a double role: it helps the reaction by infusing fresh reactants (1 and NaBH_4_) into the porous structure while expelling the freshly formed product (2) and accumulated gaseous H_2_. Manual compression did not alter the foam shape due to its high elasticity even at large deformation. This is a useful property for reusability of the material and was expected, as the purchased PUF is a material industrially optimized for repeated mechanical deformation at being soaked with aqueous solutions. Reusability was also tested *via* several complete reduction cycles of 1 (Fig. 4C). Between each run, the NPs@PUF materials were rinsed, dried, and kept in vials, and reused the following day. Excluding the first cycle, a small decrease in reaction rate can be seen from the 2nd to the 4th run. Overall, these samples were compressed more than *ca.* 2500 times and there was no observable decrease in catalytical performance. Then, a clear drop in catalytic performance happened on the 5th and last cycle. This is most probably due to deactivation of the reactive sites on the NPs by the strong adsorption of 2, which can coordinate well through the lone pairs located at the oxygen and the nitrogen atom.^65^ At first, some sites are deactivated by this adsorption, which explains the small increase in reaction time. Then, at some point, too many sites are deactivated for the reaction to proceed properly, and the NPs need to be “cleaned”. With an appropriate procedure, the catalytic particles can potentially be regenerated and recover their catalytic properties. We recently reported such a regeneration of AuNPs in sodium alginate hydrogels.^70^

Structural macroscopic intactness of the materials does not allow for concluding if the metal NPs remain attached to the support rather than being washed away. It is well conceivable that multiple cycles would decrease the NP load on the PUF support. To clarify this question, two distinct sets of thermogravimetric analysis (TGA) experiments were conducted on AuNPs@PUF. For the first test, TGA analyses were carried out before and after realizing over 100 washing cycles with water (*i.e.*, compressing the foam to expel water and subsequently re-soak it with water), resulting in an Au retention of ∼88 wt% (or ∼12 wt% of Au loss). A typical TGA thermogram for determining the amount of Au in AuNPs@PUF is presented in Fig. S4.† The Au loss can in our opinion most certainly be explained by the washing away of the biggest particles, while smaller ones (<10 nm) are stable and remain bound to PUF, as found by Domènech *et al.* for similar systems.^47^ The second test we performed consisted in applying harsher conditions to the materials by replacing water with ethanol, which is known to swell PUs.^71^ In that case, the Au retention decreases to 44 wt%, which is still fairly high and indicates again (as previously suggested by ATR-FTIR) that very small particles are covalently bound to the surface of the polymer rather than being merely physically adsorbed.

To qualitatively verify the informative value of the TGA measurements, the leftover solution from the HTS of AuNPs@PUF was treated with a 50 mM solution of the strong reducing agent NaBH_4_. Any change in color would have indicated the formation of metallic NPs and hence that a fraction of the precursor had not reacted during synthesis. For Au, no such color change was observed, indicating a complete or near-complete reaction. Indeed, the initial Au content in the precursor solution per mass of PUF (0.70%) corresponds to the amount calculated *via* TGA analysis (0.69%). For the Ag and Pd composites, the leftover solutions were slightly colored. TGA analysis confirmed that only a fraction of the initial Ag and Pd compounds per mass of PUF (0.3%) reacted, leaving the remaining amount (0.1%) to react with the reducing agent (see the ESI† for measurements and calculations).

Recently, Kuroda *et al.* have published a meta-analysis on the performance of AuNPs immobilized on polymer scaffolds for the reduction of 1.^72^ The reaction rates they summarized are comparable and of the same order as the results we report herein (∼10^−3^ s^−1^). Table 1 summarizes the key parameters used to quantify and evaluate the catalytic performances of the NPs@PUF materials synthesised in our work, including the average NP diameter *d* determined by TEM, the relative mass of metallic NPs per weight of NPs@PUF, from TGA measurements, and the apparent reaction rate *k*_app_ for the reduction of 1. Normalization of the reaction rate constant *k*_app_ to its counterpart (*k*′_app_) eliminates the effect of the size and quantity of the particles on the reaction rate. Thus, the normalized reaction rate is directly dependent on the sample's morphology and on the reaction conditions. The reaction rate *k*_app_ is divided by the NPs' total surface – the catalytic surface – and volume of the sample (eqn (S2)†). This means that more catalytically active particles, *e.g.*, the smaller PdNPs, have a bigger *k*_app_, but when taking their smaller size (*i.e.*, their larger specific surface) into account, *k*′_app_ decreases. It is interesting to notice in Table 1 that Ag and Pd display the same *k*′_app,_ while *k*′_app(Au)_ is an order smaller than these two. For the calculation of *k*′_app(Au)_, we exclusively used the small gold “seeds” since they are more catalytically active. However, there are two distinct size populations that need to be considered, as shown by electron microscopy. From electron microscopy, it is not possible to assign a meaningful weight percentage to each population. Hence, we calculated *k*′_app_ only with the diameter of the “seeds”, thus artificially decreasing the normalized reaction rate, and underestimating the reaction rate for the catalytically active AuNPs. By expecting *k*′_app(Au)_ = 10*k*′_app(Pd)_, we can actually extract an estimate of the ratio of Au_“seeds”_:Au_“big NPs”_, which is *ca.* 1 : 1 by weight, *i.e.*, ∼1000 : 1 by number (see the ESI†). By knowing the expected *k*′_app_, it is thus possible to estimate the weight fraction of the two gold populations.

We next explored the HTS of more complex, dual metal NPs with PUF. Therefore, the precursors for both Au and Pd (*i.e.*, HAuCl_4_ and Na_2_PdCl_4_) were dissolved in water and subjected to HTS in an autoclave (0.25 mM, *t*_R_ = 3 h, *T*_R_ = 120 °C) with the PUF (Fig. S5†). Two different populations were observed by TEM and TEM/EDX: Pd-rich smaller particles ∼6 nm (Fig. S6†) and big faceted particles of ∼130 nm diameter and consisting of (from EDX) circa equimolar amounts of Au and Pd. Exploratory catalytic characterization was performed as well, yielding inconsistent reaction rates (Fig. S7†), which is probably due to the compositional inhomogeneity of the particles.

**Fig. 5:**
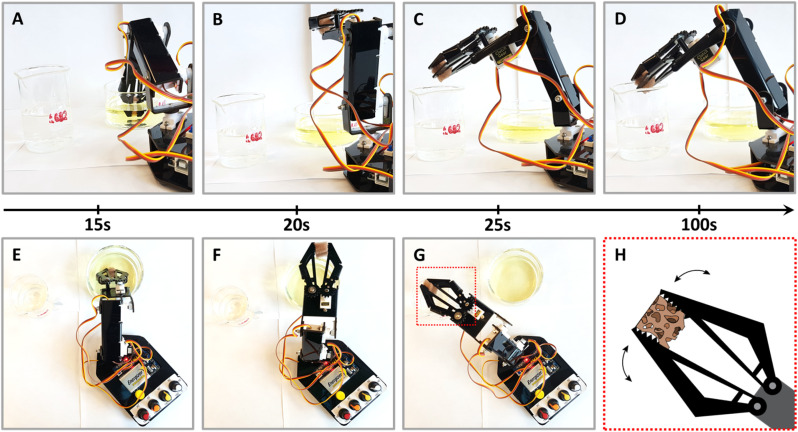
Experimental setup using a robotic arm with a claw for the continued reaction of the NPs@PUF with sequential shots of a complete reaction cycle. (A) 0–15 s, loading of the reactants – 4-NP and NaBH_4_ – by compressing the foam; (B) 15–20 s, removing the PUF from the reactant's beaker; (C) 20–25 s, transferring the sponge above the product's beaker (*i.e.*, 4-aminophenol); and (D) 25–100 s, waiting time to ensure completion of the reduction and unloading of the products. (E–G) Overhead view of shots (A–C). (H) Scheme of the highlighted box in panel (G) showing how the claw compresses and empties the PUF.

Finally, towards practical applications, we explored the use of NPs@PUF materials in a semi-automated fashion, specifically, by using a robotic arm sporting a claw. We are convinced that NPs@PUF could be of major interest in research lab-scale automation of organic synthesis.^73^ The setup is presented in Fig. 5 (see also the supplied video file “Robotic_arm_ESI.mp4” of the experiment, accelerated 32×) and illustrates the versatility and robustness of the developed materials. In essence, the robotic arm grabs and compresses the NPs@PUF to load, from a beaker, the solution containing 1 to be reduced, then moves laterally to another beaker and releases the solution now containing the product 2. Fig. 5 illustrates how a single 1 cm^3^ AuNPs@PUF was able to effectively reduce 20 mL of a solution of 1 (0.15 mM) with NaBH_4_ (25 mM). Over 25 cycles were realized, including a ∼1 min waiting time when the AuNPs@PUF is filled with the solution to ensure complete reduction. Complete conversion of 1 to 2 is demonstrated by the significant change in color of the resulting solution – from yellow (indicative of 1) to a colorless solution (indicative of an almost complete conversion to 2). This setup can easily be incorporated as a step in an effort to automatize parts of a more complex organic synthesis.

In conclusion, this work presents a new green and low-cost method to synthesize highly porous polymer monoliths covered with strongly anchored catalytic noble metal nanoparticles – materials of significant interest for fine chemical synthesis under mild conditions in aqueous medium, in batch or continuous-flow processes. The synthesis is realized under mild hydrothermal conditions (120 °C, 3 h) and only requires the noble metal precursor salts, polyurethane foam, and water. Full conversion of M^*x*+^ to metal NPs is achieved for Au and Ag, while Pd^2+^ is not fully converted to Pd^0^ under the used reaction conditions. Under hydrothermal conditions, the polyurethane foams act both as NP supports, and reducing agent, eliminating the need for strong reducing (and often toxic) chemicals. The commercial availability at low cost, the mechanical features and permanent macroporosity, the chemical stability, and the reusability are all important features of the PUFs that are retained in the resulting NPs@PUF materials, and support their applicability. Polyurethane foams, thanks to the urethane moieties, allow for the stable bonding of NPs at the surface of the PUFs, as ATR-FTIR data suggest. This strong bonding allows material reuse, as demonstrated with the model reaction of reducing *p*-nitrophenol to *p*-aminophenol in aqueous solution. Overall, the methodology we present is versatile and compatible with several NP chemistries, demonstrated herein for Au, Ag, Pd, and mixed AuPd NPs. The various NPs@PUF have been successfully applied in lab-scale catalysis. In principle, NPs@PUF materials could be quite interesting for industrial applications, especially with respect to the simplicity and greenness of the synthesis. However, to this end, scaling up studies are necessary, especially considering and eliminating the transport phenomena limitations that will arise in bigger PUFs during synthesis. The NPs@PUF materials show fast reaction kinetics, comparable to state-of-the-art catalysts, in the model reduction reaction. We furthermore demonstrated that the materials can be employed as reusable catalyst materials in a semiautomated fashion. Based on these results, in future work, we plan to more closely study how nucleation and growth of NPs occurs under various hydrothermal conditions (*e.g.*, *T*_R_, *t*_R_) and various precursor concentrations (including dual metal solutions), in order to better understand and control how to synthesize such materials. Furthermore, we intend to study in-depth the catalytic properties of these materials, with the ultimate goal to further enhance and fine-tune their catalytic properties.

## Author contributions

OG, NV, and MMU designed this project. MMU supervised this project. OG wrote the first draft, and the final manuscript was written by OG, NV, and MMU. OG performed all syntheses and characterizations, except for SEM and TEM, which were performed by AL and JB, respectively. All authors have contributed to data analysis and writing of the manuscript.

## Conflicts of interest

There are no conflicts to declare.

## Supplementary Material

TA-011-D2TA09405C-s001

TA-011-D2TA09405C-s002
