# Supplementary material for: Urethane functions can reduce metal salts under hydrothermal conditions: synthesis of noble metal nanoparticles on flexible sponges applied in semi-automated organic reduction
Source: J Mater Chem A Mater. 2023 Feb 24;11(24):12703–12. doi: 10.1039/d2ta09405c (PMC10281335; doi:10.1039/d2ta09405c)
Supplement: TA-011-D2TA09405C-s001 [file TA-011-D2TA09405C-s001.pdf]

Electronic Supplementary Material (ESI) for Journal of Materials Chemistry A.
This journal is © The Royal Society of Chemistry 2023

Supporting information

Urethane functions can reduce metal salts under hydrothermal conditions: Synthesis of noble metal nanoparticles on flexible sponges applied in semi-automated organic reduction

Olivier Gazil,[a,b] Johannes Bernardi,[c] Arthur Lassus,[b] Nick Virgilio [b] and Miriam M. Unterlass *[a,d]

[a.] Universität Konstanz, Department of Chemistry, Solid State Chemistry, Universitätsstrasse 10, D-78464 Konstanz, Germany. E-mail: miriam.unterlass@uni-konstanz.de.

[b.] CREPEC, Department of Chemical Engineering, Polytechnique Montréal, C.P. 6079 Succursale Centre-Ville, Montréal, Québec H3C 3A7, Canada

[c.] University Service Centre for Transmission Electron Microscopy, Vienna University of Technology, Wiedner Hauptstrasse 8-10/137, A-1040 Vienna, Austria.

[d.] Center for Molecular Medicine of the Austrian Academy of Sciences (CeMM), Lazarettgasse 14, AKH BT25.3, 1090 Vienna, Austria

I. Methodology

II. Supplementary Figures

III. Supplementary Equations

IV. Supplementary Results

V. References

I. Methodology

Materials

Gold(III) chloride trihydrate ($HAuCl_4·3H_2O$), silver nitrate ($AgNO_3$) and sodium tetrachloropalladate(II) (Na_2PdCl_4) were all purchased from Sigma-Aldrich (reference numbers 520918, 792276, 205818). 4-nitrophenol (4-NP) and sodium borohydride ($NaBH_4$) were purchased from Fischer Scientific (11456967 and 11359359). All chemicals were of reagent grade and were used as received without further purification. The commercial polyurethane foam was purchased at a local branch of the hardware store OBI.

Preparation of the metal nanoparticles (NPs)@polyurethane foam (PUF) hybrids

The typical preparation of NPs@PUF hybrids started from commercial polyurethane foams shaped into 1.1 cm edge cubes. Each cube was plunged into 5 mL of a 0.25 mM metallic precursor ($HAuCl_4$, $AgNO_3$ or Na_2PdCl_4) solution in a custom-made glass vial, then placed in an autoclave at 120°C for 3 h (at an approximate pressure of 2 bar). The resulting materials were then washed several times with distilled water and dried at room temperature.

Microwave reactor synthesis of AuNPs@PUF

The synthesis was performed in an Anton Paar Monowave 450 in a G10 glass vial. The PUF cube was plunged into 5 mL of a 0.25 mM metallic precursor and heated at 120 °C. The heating rate was as fast as possible (using maximum power of the Monowave – 850 W) to reach the desired 120 °C temperature under constant stirring. Then, the sample was kept for 30 min at this temperature with no stirring.

Optical microscopy characterization

Observations were performed with a reflected light microscope (Leica M125 optical microscope) at 32X magnification.

Attenuated total reflection Fourier transform infrared spectroscopy (ATR-FTIR)

ATR-FTIR measurements were realized with a Bruker Tensor 27 instrument working in ATR MicroFocusing MVP-QL equipped with a diamond crystal, over a range of 450 cm^{-1} to 4000 cm^{-1}. The dried samples were used directly with no further preparation on the crystal.

X-ray diffraction (XRD)

XRD experiments were realised on a PANalytical X'Pert Pro multipurpose diffractometer (MPD) in Bragg Brentano geometry, operating with a Cu anode at 40 kV, 40 mA. Scans from 5° to 90° over 1 h were performed for the dried samples. This high measurement time was necessary to obtain distinct peaks for the NPs above the PUF background signal.

Transmission electron microscopy (TEM) characterization

TEM micrographs were obtained with a FEI Tecnai F20 S/TEM equipped with a GATAN Rio-16 camera. The observations were realized at 200 kV using brightfield and darkfield modes. Samples were prepared by filling the pores with an epoxy resin, followed by ultramicrotomy (~100 nm thickness) at room T and deposition on TEM grids. An example of a AuNPs@PUF sample encased in epoxy and observed by SEM is displayed in Figure S8. NPs sizes were determined by analysing 70+ particles with the image analysis software ImageJ.

Thermogravimetric analysis (TGA) of PUF hybrids

The NPs content in the PUF hybrids was measured with a Perkin Elmer TGA 8000. Approximately 2 mg of sample were heated up to 450 °C at a rate of 10 °C/min under a constant nitrogen flow (20 mL/min). Three specimens sampled on different PUF hybrids were analysed for each type of metal NPs. Furthermore, the degradation of the polymer phase led to the accumulation of by-products onto the crucible's hook. For this reason, the apparatus' weight was reinitialized at the beginning of each analysis. The instrument has a sensitivity of 0.1 µg.

Characterization of the catalytic properties

The catalytic properties were characterized by using the model reduction reaction of 4-nitrophenol to 4-aminophenol (4-AP), catalysed on a noble metal NPs surface, as described by Pradhan et al.[1] The extent of reaction was recorded by UV-vis with a Thermo Scientific™ NanoDrop™ One spectrophotometer at 400 nm. Monitoring at this wavelength was necessary to measure the relative absorption of 4-NP, which can be converted to the relative concentration of 4-NP ($[4\text{-NP}]/[4\text{-NP}]_{t=0}$) using a calibration curve. The apparent reaction rate was measured by fitting an exponential curve to the data – the relative concentration plotted versus time. In a typical experiment, 1.5 mL of 4-NP (0.3 mM) was added to a 45 mm tall quartz cuvette followed by 1.5 mL of $NaBH_4$ (50 mM). Note that nitrogen was initially bubbled for 30 min in the solutions to minimize the presence of dissolved oxygen species, which limits the reaction.[2] Afterwards, a sample of metal NPs@PUF (approximate volume of 0.2 cm^3) was added to the cuvette, marking the beginning of the reaction performed at room temperature. While the tests were carried out, the NPs@PUF samples were compressed with tweezers to manually mix the reaction medium. Finally, when the distinct yellow coloration of 4-NP had mostly disappeared (meaning reaction completion), the samples were removed, washed with distilled water, and dried for subsequent testing. At least 3 experiments were realized per reported experimental point.

II. Supplementary Figures

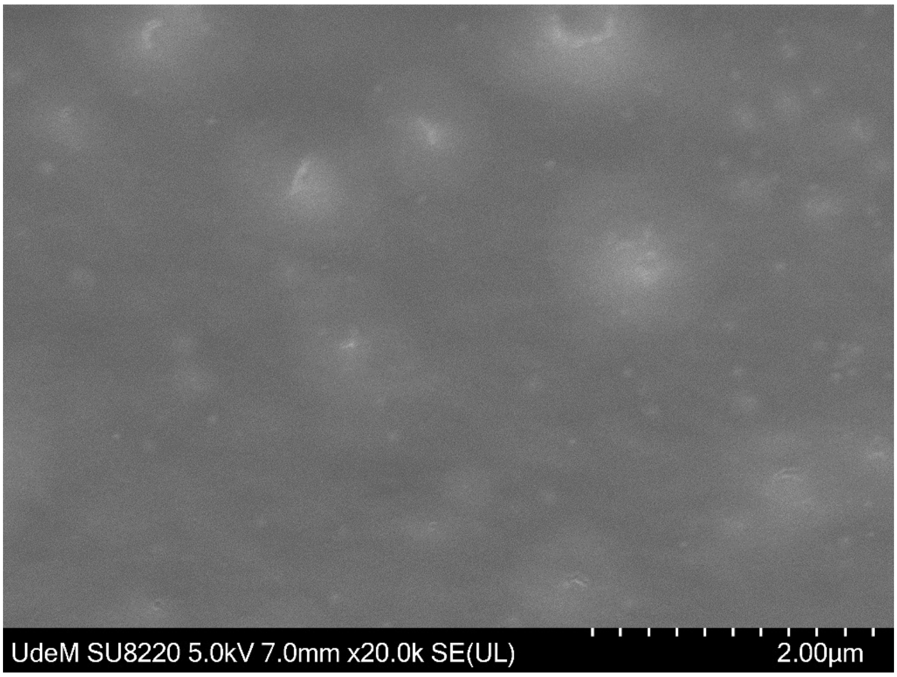

Figure S1. SEM micrograph of the starting pristine PUF at higher magnification showing no apparent nano/microstructures on the surface, as a comparison point for the NP@PUF synthesis.

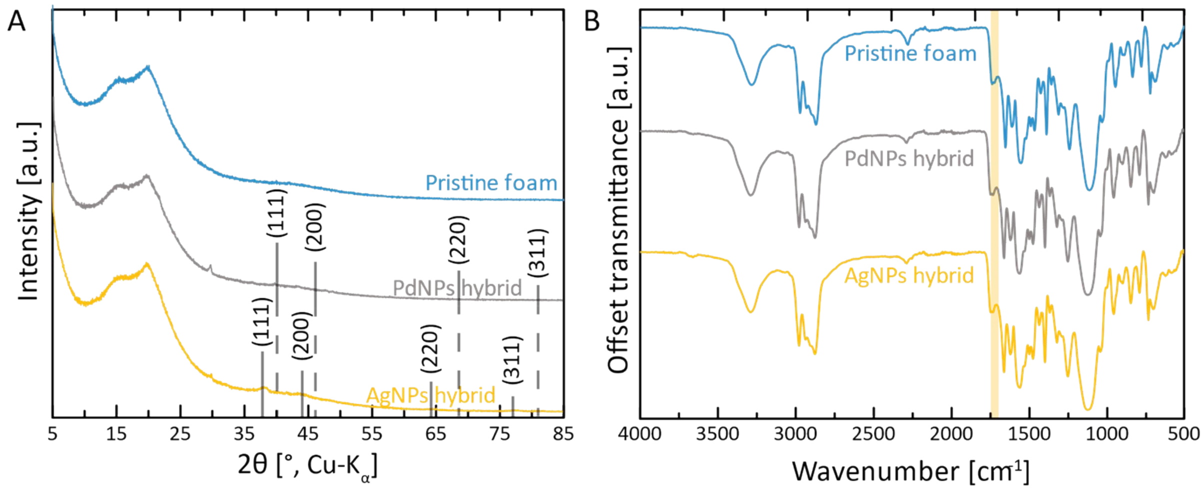

Figure S2. XRD (A) and IR (B) spectra of AgNPs and PdNPs polyurethane foam hybrid nanocomposites compared to the pristine foam. B) The respective C=O mode associated with an adsorption of around 1720 cm^{-1} is highlighted in yellow for the various nanocomposites.

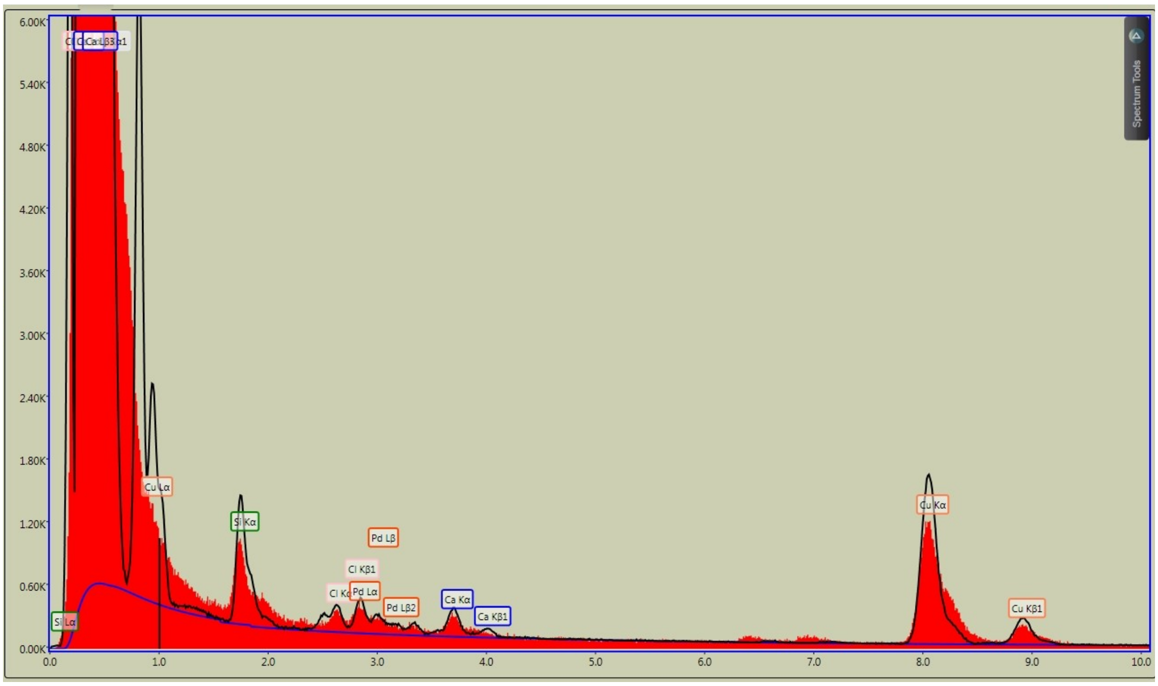

Figure S3. Complete EDX spectrum of the region highlighted in a white box in the PdNPs composites' TEM micrograph presented in Figure 3D. On the y-axis, the total occurrence of X-ray with a specific energy, represented on the x-axis (keV).

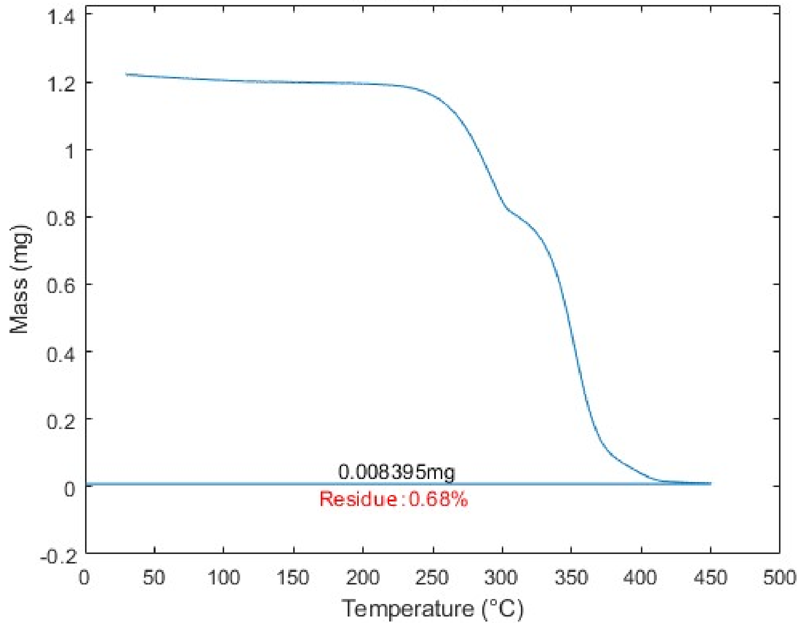

Figure S4. TGA thermogram of a typical sample of AuNPs@PUF composite completely burned to extract the amount of AuNPs (indicated as residue in the graph) per mass of hybrid composite.

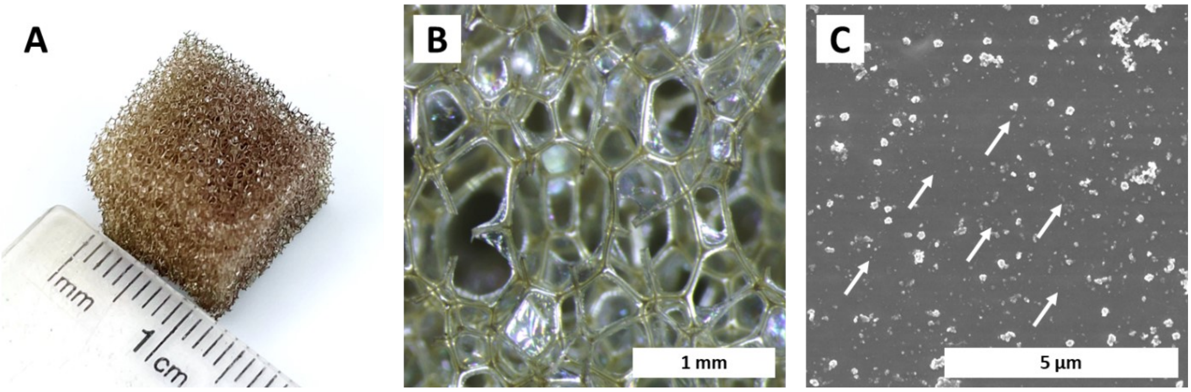

Figure S5. Macroscopic view (A) and its associated optical micrograph (B) of AuPdNPs@PUFs synthesized with $HAuCl_4$ and Na_2PdCl_4 (0.125 mM, 3 h, 120 °C). C) SEM micrographs of AuPdNPs@PUFs. Note the two different types of particles: smaller darker particles (some are indicated with white arrows) and bigger brighter particles corresponding respectively to the populations observed in Fig.S6A and Fig.S6B.

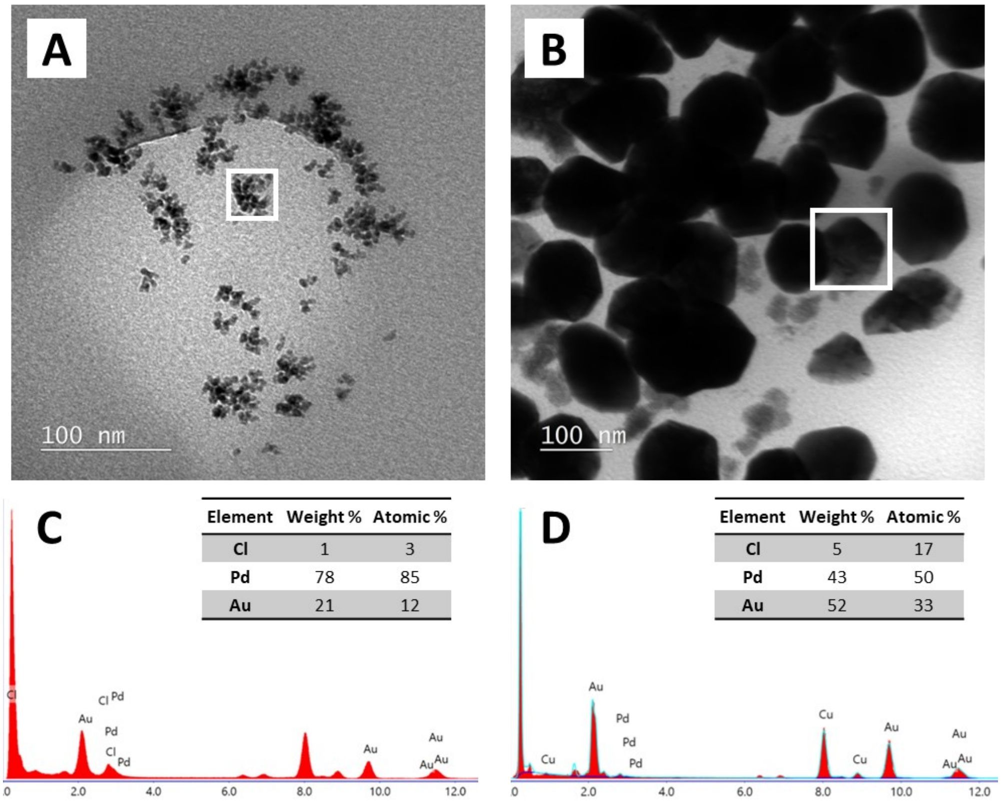

Figure S6. TEM micrographs (A-B) of AuPdNPs@PUF with corresponding EDX spectrum of the framed region (C-D). Note the two different morphologies of the particles for the synthesis of Au-Pd hybrid particles. C-D) Respective EDX spectra of A-B, highlighting the compositions in Au, Pd and Cl.

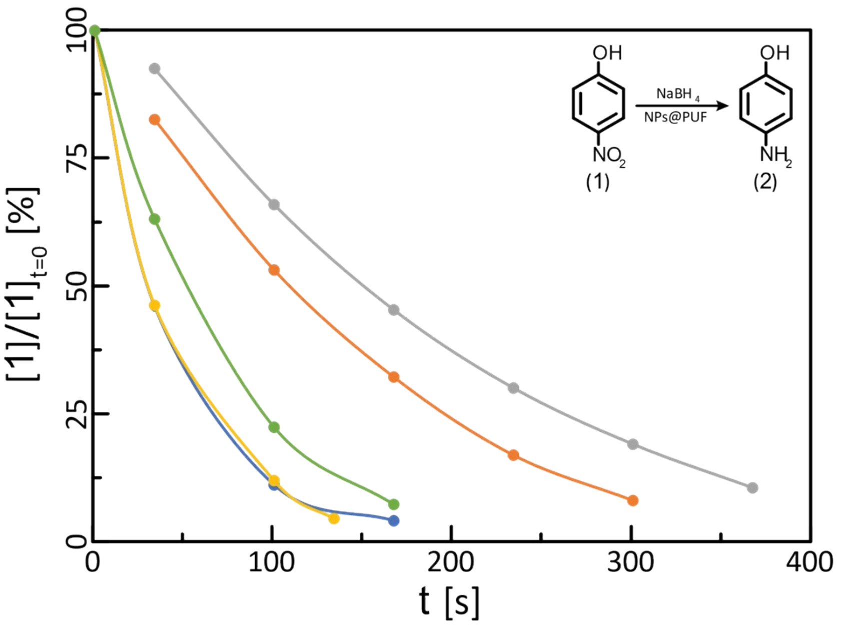

Figure S7. Plot of the relative concentration of 4-NP versus time for the determination of the reaction rate constant (N = 5) for AuPdNPs@PUF.

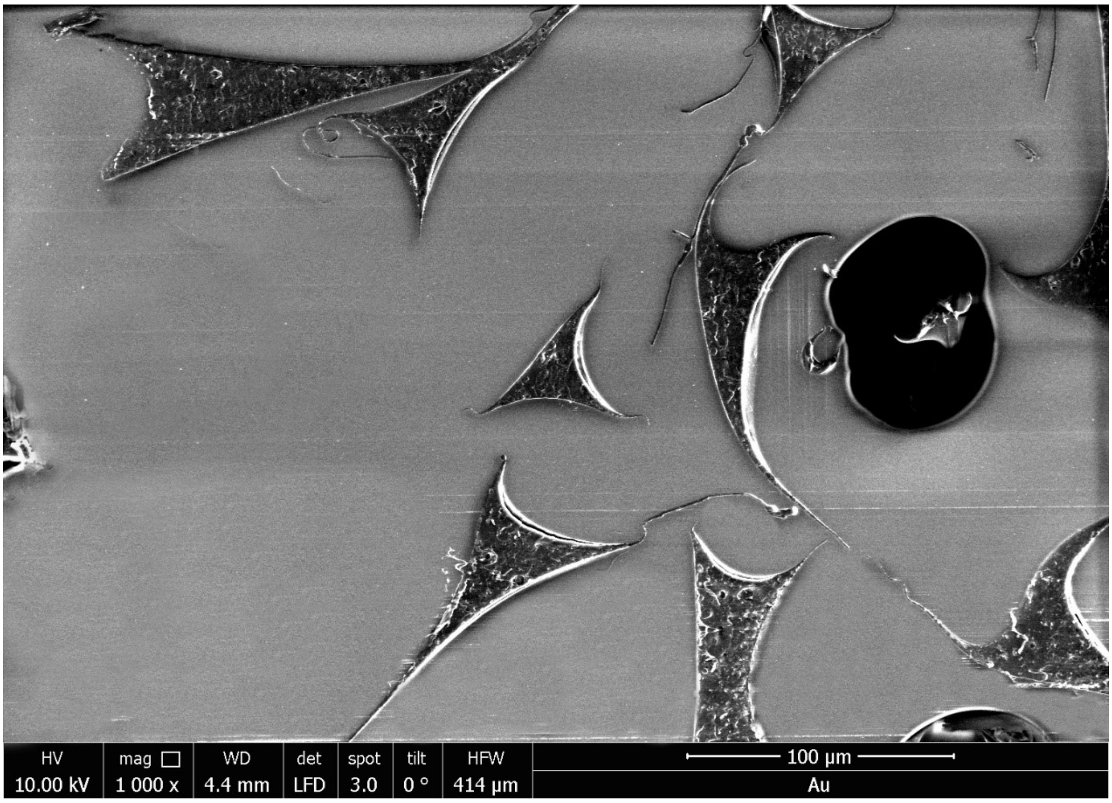

Figure S8. SEM of PUF encased in epoxy for the preparation of ultramicrotomed slices for TEM.

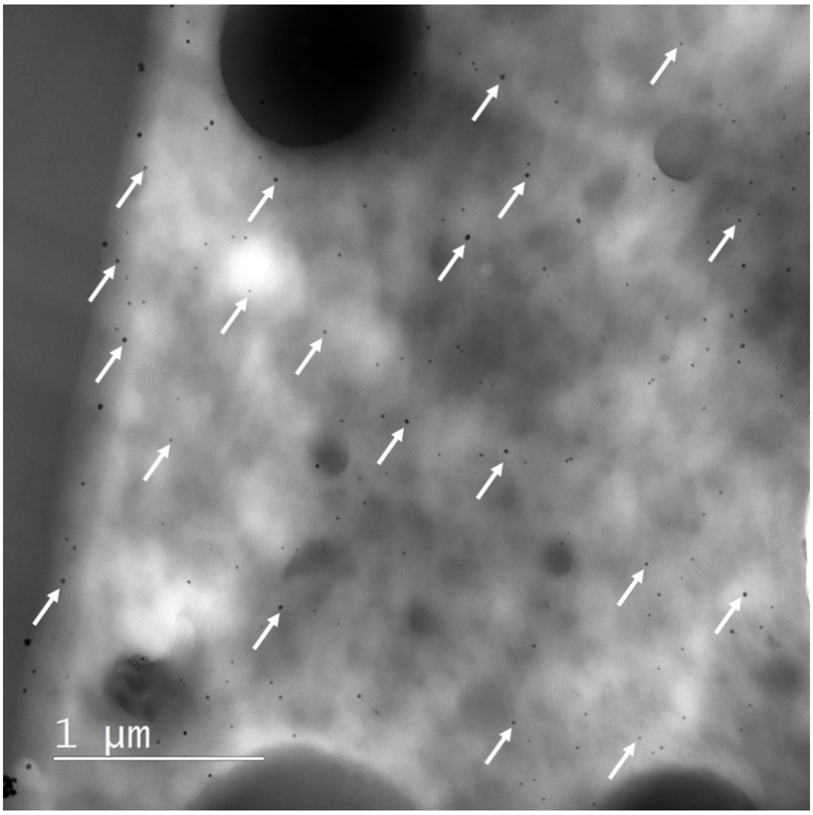

Figure S9. TEM micrograph of the surface of a PUF's strut (microtomed along its axis), highlighted by the high presence of silver NPs (some indicated with arrows).

III. Supplementary Equations

$$\tau = \frac{K\lambda}{\beta cos\theta} \quad \text{(S1)}$$

τ is the crystallites size (nm), K is the shape factor (has typically a value of 0.9, when the crystallites are considered spherical), λ is the X-ray wavelength (nm), θ is the Bragg angle (radians) and β is the full width at half maximum (FWHM) when subtracting the line broadening caused by the instrument (radians).

$$k_{app}^{'} = \frac{k_{app}}{S_{NPs}/V_{sample}} \quad \text{(S2)}$$

k'_{app} is the normalized reaction rate ($s^{-1}m^{-2}L$), k_{app} is the reaction rate (s^{-1}), S_{NPs} is the combined surfaces of all the particles contained in the volume of the sample used for catalysis (m^2), V_{sample} (L). S_{NPs} is calculated with the following equation.

$$S_{NPs} = \frac{\%^{m}/_{m}{}^{NPs} * m_{sample}}{\rho_{Au} * {}^{3}/_{2} d_{NPs}} \quad \text{(S3)}$$

% m/m_{NPs} is the weight percentage of nanoparticles in the PUF hybrids, m_{sample} is the mass of the sample used for catalysis (g), ρ_{Au} is the density of gold (g/m^3) and d_{NPs} is the average diameter of the nanoparticles determined via statistical analysis on TEM micrographs (m).

IV. Supplementary Results

Comments on TEM observations

A 100 nm thick sample of a 60 µm PUF means that, at best, it will be possible to observe particles over 0.2% of the microtomed surface – since they should be exclusively on the surface. However, in rare cases where a sample is taken on the fiber surface (along its axis), the whole microtomed surface would display particles (e.g. Fig.S9). Larger particles, seldom present on the sampled surface considering the thickness of a slice, mean the statistical significance of the population's size will decrease. In other words, the larger the particles, the harder the measurement to obtain a precise size distribution and average size value. The continuity of the line drawn by the particles supports this statement (it is noteworthy to mention that this line represents the polymer surface): Fig. 3D has a nearly continuous line of PdNPs due to the presence of a greater number of small particles, as opposed to Fig. 3A that has a broken line of AuNPs in many places, with larger particles.

Determination of the crystallite size from XRD data

-By Scherrer's equation:

We suppose a circular shape for the crystallites (K = 0.9), we have a wavelength of 0.15406 nm for Cu-K_α radiation, a full width at half maximum of 0.77° for the 38.1° reflex and a line broadening caused by the instrument of approximately 0.06°.

$$\beta = FWHM - \beta_{broadening} = (0.77° - 0.06°) * \frac{\pi \ rad}{180 \ °} = 0.0123$$

$$\tau = \frac{K\lambda}{\beta cos\theta} = \frac{0.9 * 0.15406 \, nm}{0.0123 * \cos\left(38.1 * \frac{\pi \ rad}{180 \ °}\right)} = 11.9 \, nm$$

-By Rietveld refinement:

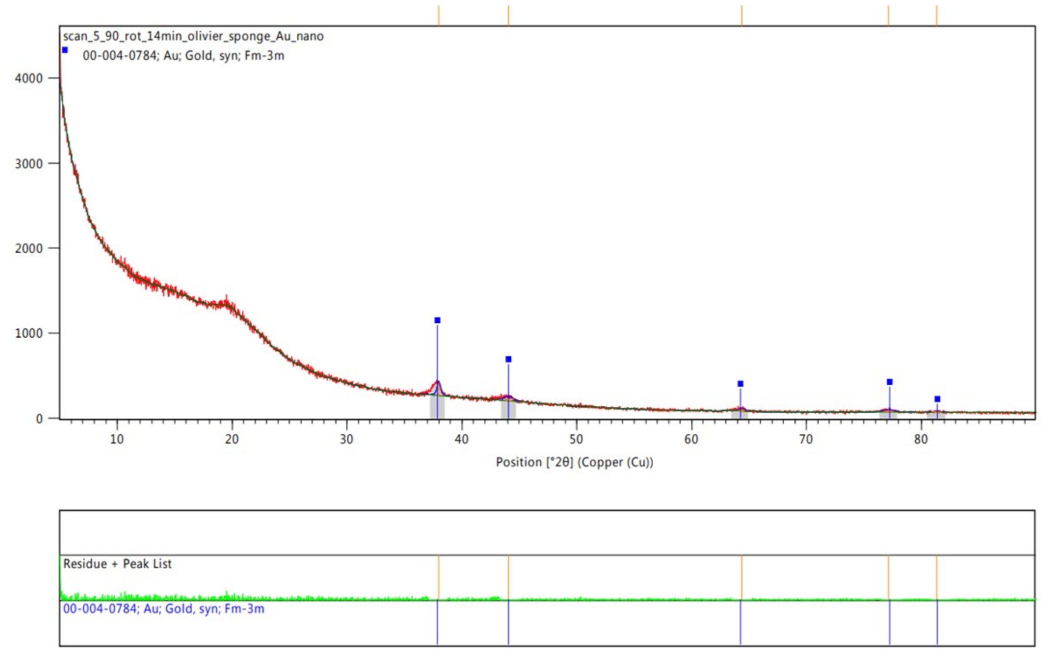

Figure S10. Peak attribution and integration done on HighScore Plus (powder diffraction analysis software) to determine crystallite size via the Rietveld refinement technique (yielding a crystallite size of 14.5 nm).

$k'_{app(Au)}$ discrepancy – w% assignation for the two gold populations

Considering the seeds population as population #1 and the bigger particles as population #2, we have the average diameter of the two population as follow: d_1 = 3 nm and d_2 = 30 nm. From $k'_{app(Ag)}$ and $k'_{app(Pd)}$ (where $k'_{app(Ag)} \approx k'_{app(Pd)}$), we expect the value of $k'_{app(Au)}$ to be roughly equivalent to ~4E-03 $s^{-1}m^2L$, which will be used to estimate the weight fraction of each population.

$$k'_{app(Au)} = \frac{k_{app(Au)}}{S_{NPs}/V_{sample}}$$

We know that the total surface of the two populations of gold particles is equal to:

$$S_{NPs} = \frac{\%^{m}/_{m^{NPs}} * m_{sample}}{\rho_{Au} * {}^{3}/_{2}} * \left(\frac{w\%_1}{d_1} + \frac{(1 - w\%_1)}{d_2}\right)$$

$w\%_1$ is the weight fraction of gold of population #1 (i.e. "seeds") and $w\%_2 = 1 - w\%_1$.

We can replace all known parameters and estimate the value of $w\%_1$:

$$4 * 10^{-3}\, s^{-1}m^{-2}L = \frac{3 * 10^{-3}\, s^{-1}}{\frac{0.024 * 10^{-3}\, g}{19.32\, g/10^{-6}\, m^3 * {}^{3}/_{2}} * \left(\frac{w\%_1}{3\, nm} + \frac{(1 - w\%_1)}{30\, nm}\right) \Big/ 0.2 * 10^{-3}\, L}$$

$$\left(1.81 * 10^7 * 10^{-9}\right) nm^{-1} = \left(\frac{w\%_1}{3\, nm} + \frac{(1 - w\%_1)}{30\, nm}\right)$$

$$5.43 = 10\, w\%_1 + 1 - w\%_1$$

$$w\%_1 = 0.49 = 49\%$$

For $k'_{app(Au)}$ to be equal to $k'_{app(Ag)}$ and $k'_{app(Pd)}$, we need to have 49w% "seeds" and 51w% of bigger particles, which means we have a weight ratio between the two population of 1:1. We can extract the number ratio of (where n is number of particles):

$$m_{Au\,1} = n_1 * V_{sphere\,1}/\rho_{Au}$$

$$m_{Au\,1} = m_{Au\,2}$$

$$n_1 * \frac{V_{sphere\,1}}{\rho_{Au}} = n_2 * \frac{V_{sphere\,2}}{\rho_{Au}}$$

$$n_1 * \frac{4}{3}\pi * \left(\frac{d_1}{2}\right)^3 = n_2 * \frac{4}{3}\pi * \left(\frac{d_2}{2}\right)^3$$

$$n_1 * (3\, nm)^3 = n_2 * (30\, nm)^3$$

$$n_1 = 1000\, n_2$$

The ratio "seeds" to big particles is 1000:1.

V. References

1 N. Pradhan, A. Pal and T. Pal, *Colloids and Surfaces A: Physicochemical and Engineering Aspects*, 2002, **196**, 247-257.

2 E. Menumerov, R. A. Hughes and S. Neretina, *Nano Lett*, 2016, **16**, 7791-7797.
